# Supplementary material for: DMRforPairs: identifying Differentially Methylated Regions between unique samples using array based methylation profiles
Source: BMC Bioinformatics. 2014 May 15;15:141. doi: 10.1186/1471-2105-15-141 (PMC4046028; doi:10.1186/1471-2105-15-141)
Supplement: Additional file 2 — DMRforPairs output for the comparison of A431-MCF7 and NA17018-NA17105. Please start from the HTML files in each folder. Available via the BMC Bioinformatics website. [file 1471-2105-15-141-S2.zip › 1394847754114233_MOESM2_ESM/A431_MCF7/figures/35512.pdf]

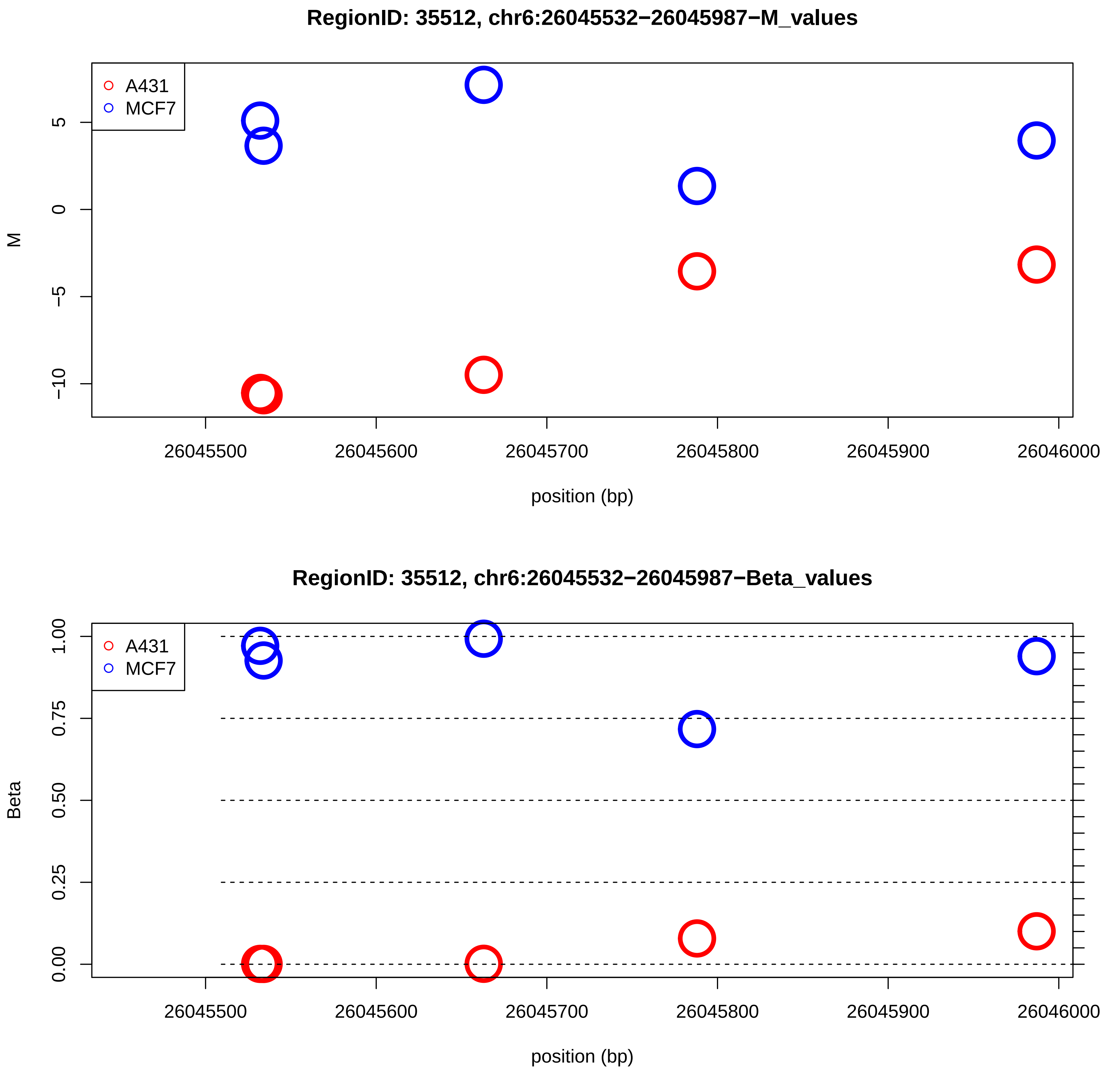
RegionID: 35512, chr6:26045532−26045987−M_values
A431
MCF7
M
5
0
−5
−10
26045500
26045600
26045700
26045800
26045900
26046000
position (bp)
RegionID: 35512, chr6:26045532−26045987−Beta_values
A431
MCF7
Beta
1.00
0.75
0.50
0.25
0.00
26045500
26045600
26045700
26045800
26045900
26046000
position (bp)

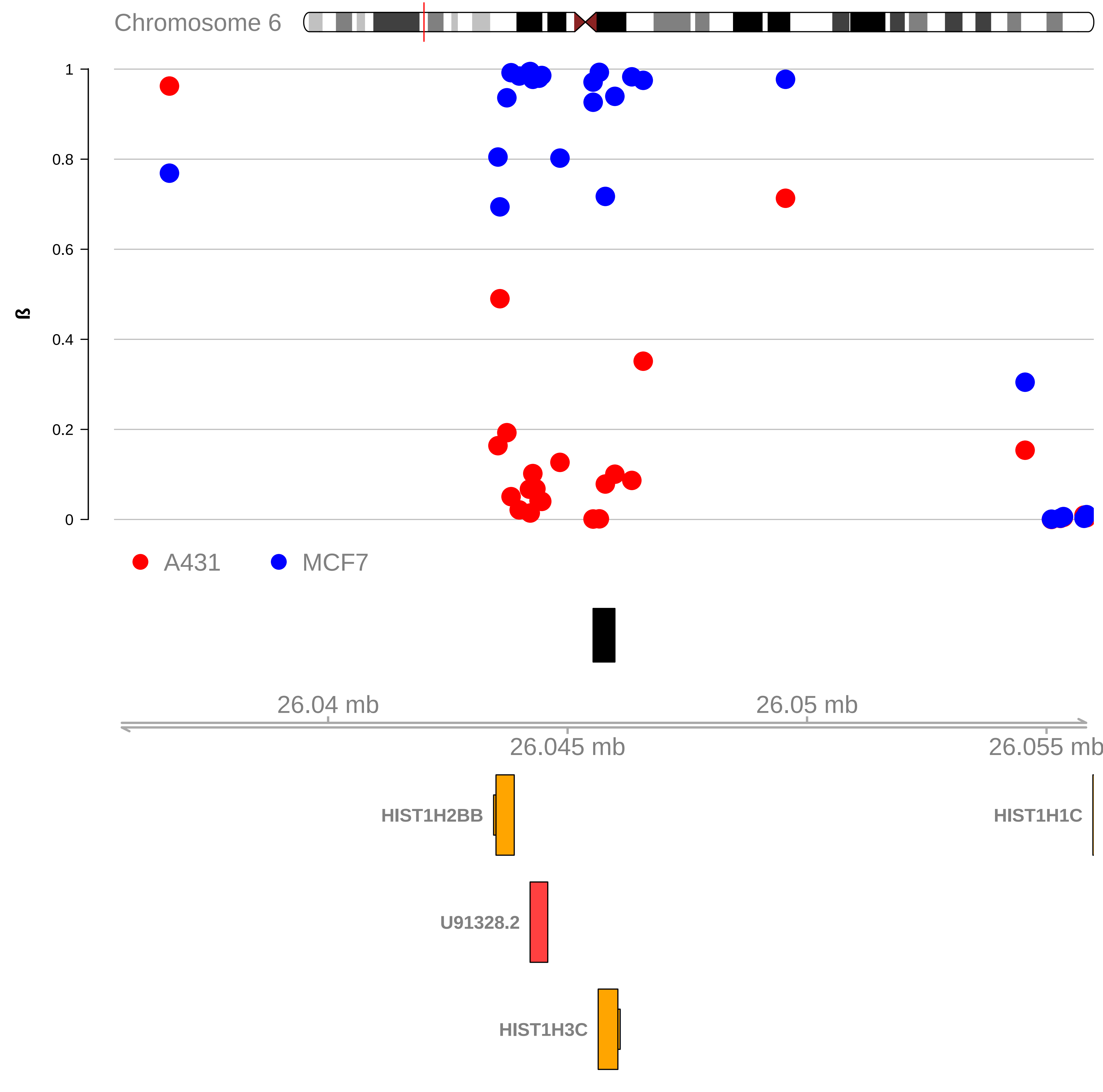

Chromosome 6
1
0.8
0.6
0.4
0.2
0
ß
A431
MCF7
26.04 mb
26.05 mb
26.045 mb
26.055 mb
HIST1H2BB
HIST1H1C
U91328.2
HIST1H3C
